# Macrophage-Secreted S100A4 Supports Breast Cancer Metastasis by Remodeling the Extracellular Matrix in the Premetastatic Niche

**DOI:** 10.1155/2022/9895504

**Published:** 2022-04-20

**Authors:** Yana Qi, Tingting Zhao, Ranran Li, Mingyong Han

**Affiliations:** Cancer Therapy and Research Center, Shandong Provincial Hospital, Cheeloo College of Medicine, Shandong University, Jinan, 250021 Shandong, China

## Abstract

Metastasis is the major cause of cancer-related mortalities. A tumor-supportive microenvironment, also known as the premetastatic niche at secondary tumor sites, plays a crucial role in metastasis. Remodeling of the extracellular matrix (ECM) is essential for premetastatic niche formation, especially for circulating tumor cell colonization. However, the underlying molecular mechanism that contributes to this effect remains unclear. Here, we developed a lung metastasis model with 4T1 breast cancer cells and found that the metastasis critically depended on the early recruitment of macrophages to the lung. Disruption of macrophage recruitment reduced fibroblast activation and lung metastasis. Furthermore, we identified the secreted protein S100A4, which is produced by M2 macrophages and participates in fibroblast activation and ECM protein deposition via the ERK signaling pathway. Collectively, these results indicate that recruiting S100A4-expressing inflammatory macrophages plays a vital role in ECM remodeling in the premetastatic niche and may act as a potential therapeutic target for breast cancer lung metastasis.

## 1. Introduction

The second leading cause of mortality among women is breast cancer [1]. However, a large number of these deaths occur as a result of metastasis. Breast cancer metastasizes to the lungs in most cases [2, 3], but the mechanisms remain largely unclear [4–6]. Recently, increasing evidence has suggested that primary tumors can trigger the formation of microenvironments in distant organs that are favorable for the survival and outgrowth of malignant cells before their arrival at these sites. This supportive microenvironment is termed the premetastatic niche. Formation of the premetastatic niche is essential for the extravasation and colonization of circulating cancer cells in secondary organs [7–10]. Thus, targeting the cells and molecules that participate in the formation of the premetastatic niche may prevent cancer metastasis.

The formation of the premetastatic niche is characterized by extracellular matrix remodeling, immunosuppression, inflammation, lymphangiogenesis, organotropism, and vascular hyperpermeability [11]. Remodeling of the ECM is essential for premetastatic niche formation, and fibrosis is a result of ECM remodeling [12, 13]. In some way, a tumor can be regarded as a fibrotic organ containing cancer cells. Tumor-associated fibrosis is a chronic inflammatory response that is characterized by ECM protein deposition, angiogenesis, and fibroblast activation. Fibroblasts in healthy lungs are quiescent, synthesizing little collagen; yet, during fibrosis, they differentiate into myofibroblasts, characterized by increased expression of *α*-smooth muscle actin (*α*-SMA) and fibroblast activation protein (FAP). Myofibroblasts play an important role in tissue remodeling and the secretion of ECM proteins such as fibronectin and type I collagen during fibrogenesis [14, 15]. The fibrotic milieu can promote tumor cell colonization [16, 17]. Chronic inflammatory responses usually cause scarring, ultimately leading to the incidence and progression of fibrosis. Inflammatory cells, such as macrophages, play a crucial role in promoting the inflammatory process [18–21]. In primary cancers, many research reports have demonstrated the role of macrophages in promoting metastatic progression. These tumor-associated macrophages (TAMs) could promote the growth of tumors via multiple mechanisms, such as initiating angiogenesis, inhibiting the immune response, and activating ECM remodeling [22–24]. However, it is still unclear on the role of macrophages at the secondary metastatic sites.

S100A4, which is also referred to as FSP-1 (fibroblast-specific protein-1), is a member of S100 EF-hand calcium-binding proteins [25, 26]. The most well-known function of S100A4 is to promote tumor metastasis [27]. In addition, it is a marker of fibroblasts in fibrotic organs [28, 29]. However, this protein cannot be used as a specific marker for fibroblasts overall. In a mouse model of kidney injury, Inoue and colleagues showed that S100A4 was coexpressed with Mac and CD68 macrophage antigens [30]. Similar research findings were reported in pulmonary and liver fibrosis [19, 31, 32]. As a result, we hypothesized that in the premetastatic lungs, S100A4 might also be produced by macrophages and be associated with the pathophysiology of fibrosis.

The extracellular signal-regulated kinase (ERK) signaling pathway is a key regulator of various cellular functions, such as proliferation, differentiation, survival, and motility [33]. ERK1/2 activation stimulates cell proliferation in general, and its dysregulation is a significant feature of many malignancies [34]. In addition, several of the S100A4-responsive cells, including cardiac myocytes [35] and human pulmonary artery smooth muscle cells [36], have been found to have the ability to activate ERK1/2. Most of S100A4's effects are presumably mediated by ERK1/2, which is involved in both survival and growth responses. Nevertheless, the possible involvement of the ERK signaling pathways in the progression of fibrosis induced by S100A4 has not been explored.

First of all, we investigated the role of macrophages in the process of ECM remodeling in the present research, particularly in the premetastatic niche. Furthermore, we defined macrophage-secreted S100A4 as an important mediator in the pathogenesis of ECM remodeling in the premetastatic niche. S100A4-expressing macrophage suppression might be an effective therapeutic method that can be used to inhibit ECM remodeling during premetastatic niche formation.

## 2. Materials and Methods

### 2.1. Cell Culture

The American Type Culture Collection supplied the mouse fibroblast cell line L929, mouse breast cancer cell line 4T1 and mouse monocytic cell line RAW264.7 (ATCC, Manassas, VA, USA). The cells were subjected to culturing at a temperature of 37°C in humidified air with 5% CO_2_ in RPMI 1640 with 10% fetal bovine serum (FBS).

### 2.2. Macrophage Activation

For the purpose of generating M1 macrophages, RAW 264.7 cells were subjected to treatment with 100 ng/ml lipopolysaccharide (LPS, Sigma) for 24 hours. They were induced to the M2 phenotype by adding IL-4 (20 ng/ml, PeproTech) and IL-13 (20 ng/ml, PeproTech) cotreatment for 24 h. Twenty-four hours following macrophage activation, conditioned medium (CM) was obtained as cell culture supernatants in serum-free 1640 media.

### 2.3. Animals

The Center for New Drug Evaluation supplied female BALB/c mice (6-8 weeks old) (Shandong University, Jinan, China). The Shandong Provincial Hospital's Committee for Ethics of Animal Experiments granted its approval for all of the experiments conducted in the present research.

### 2.4. In Vivo Studies

We developed a lung metastasis model with 4T1 tumor cells according to our previous studies [10, 37]. This model is a spontaneous metastasis breast cancer model that mimics human breast cancer patients. In brief, 1 × 10^6^ 4T1 cells were subjected to suspending in 0.1 ml of serum-free RPMI 1640 and injection into each BALB/c mouse's paired abdominal mammary glands. Similarly, PBS was administered as a control in isometric quantities. On days 0, 7, 14, and 21, five mice from each of the 4T1 and the control groups were killed by means of injecting pentobarbital intraperitoneally and PBS perfusion via the right cardiac ventricle. Clodronate liposomes (CL, 100 mg/kg) were delivered 24 hours intravenously after the 4T1 cells were implanted into the mammary fat pad of BALB/c mice, followed by recurrent injections of 50 mg/kg each fourth day, as provided in a previous report [38]. The control group was intravenously injected with isometric amounts of PBS liposomes (PL).

### 2.5. Histopathology Analysis

The lungs were preserved in 4% paraformaldehyde overnight, followed by embedding in paraffin. They were then sliced into 4 *μ*m thick slices for the purpose of performing H&E staining. Histopathological examination was performed on an average of three mice per group at each experimental time point. Two expert pathologists who were blinded to the research groups captured and analyzed all slices. H&E staining was carried out for the purpose of assessing lung inflammation in accordance with the criteria proposed in previous studies [39, 40].

### 2.6. Immunofluorescence Staining

In 6-well plates with coverslips, L929 cells were seeded. Subsequently, the coverslips were fixed in 4% paraformaldehyde for twenty minutes, followed by permeabilization with 0.1% Triton X-100. Following 30 minutes of blocking with 5% BSA, the coverslips were subjected to incubation overnight at 4°C with rabbit anti-*α*-SMA (1 : 100, ab5694, Abcam, Cambridge, UK) or mouse anti-fibronectin (1 : 100, sc-59826, Santa Cruz, Dallas, Texas, USA) in combination with additional incubation using the secondary fluorescence-labeled antibodies. F4/80, *α*-SMA, and fibronectin were subjected to immunofluorescently staining in tissue slices from mouse lungs with the aid of a mouse anti-F4/80 antibody (1 : 100, sc-377009, Santa Cruz, Dallas, Texas, USA), a rabbit anti-SMA antibody (1 : 200, ab5694, Abcam, Cambridge, UK), and a mouse anti-fibronectin antibody (1 : 200, sc-59826, Santa Cruz, Dallas, Texas, USA). M2 macrophages and lung paraffin sections were exposed to treatment overnight at 4°C with a combination of rabbit anti-S100A4 monoclonal antibody (ab197896, Abcam, Cambridge, UK) at a 1 : 100 dilution and mouse anti-CD206 antibody (sc-58986, Santa Cruz, Dallas, Texas, USA) at a 1 : 50 dilution. This procedure was followed by exposure to a mixture for 1 hour at room temperature treatment with the secondary fluorescence-labeled antibodies. Ultimately, the samples were incubated with DAPI for nuclear staining and visualized under a light microscope (DM500; Leica, Mannheim, Germany).

### 2.7. RNA Interference and Cell Transfection

Negative control sequence (NC siRNA) and S100A4 siRNA were designed by Genomeditech (Shanghai, China). The target sequences were as follows: S100A4 siRNA: 5′-GAUGUGCAAUGAAUUCUUU-3′; NC siRNA: 5′-UUCUCCGAACGUGUCACGU-3′. M2 macrophages were transfected with siS100A4 in the upper chambers. Lipofectamine MAX (Invitrogen, Camarillo, CA, USA) was utilized as the transfection reagent in accordance with the manufacturer's instructions. Then, the transfected macrophages were cocultured with fibroblasts for 48 h in a Transwell system (0.4 mm PET, 4.5 cm^2^, Millipore).

### 2.8. RT-qPCR

Total RNA was extracted with the aid of the TRIzol Reagent (Invitrogen, CA, USA) in accordance with the guidelines provided by the manufacturer. Reverse transcription was carried out in a 10 L reaction system utilizing Evo M-MLV RT Premix (Accurate Biotechnology, Hunan, China) as specified by the manufacturer. Real-time PCR was performed for the purpose of amplifying the cDNA using SYBR Green Pro Taq HS Premix (Accurate Biotechnology) and the Light Cycler 480 equipment (Roche Diagnostics, Basel, Switzerland). Relative quantification was performed using the 2^−△△Ct^ method. The relative expression levels of the target genes were standardized to GAPDH, and every sample was repeated thrice. The forward (F) and reverse (R) primers used were as follows: GAPDH-forward (F), TGGCCTTCCGTGTTCCTAC; GAPDH-reverse (R), GAGTTGCTGTTGAAGTCGCA; Acta2-F, TTCGTGACTACTGCCGAGC; Acta2-R, GTCAGGCAGTTCGTAGCTCT; FN-F, ACCTTGATCTCCCAAGCACG; FN-R, CGTCAGGTGCTGTAGTCTGT; FAP-F, GGATGGGCTGGTGGATTCTT; FAP-R, CCTCCCACTTGCCACTTGTA; CD206-F, ACGACAATCCTGTCTCCTTTGT; CD206-R, CAGATATGCAGGGAGTCACC; Arg-1-F, GGAACCCAGAGAGAGCATGA; Arg-1-R, TTTTTCCAGCAGACCAGCTT; S100A4-F, TCAGCACTTCCTCTCTCTTGG; S100A4-R, AACTTGTCACCCTCTTTGCC; and Inos-F, TCTAGTGAAGCAAAGCCCAACA; Inos-R, CTCTCCACTGCCCCAGTTTT.

### 2.9. Western Blot Analysis

RIPA lysis buffer that contained a protease inhibitor cocktail (Beyotime Biotechnology, Shanghai) was utilized to lyse cells and lung tissues for thirty minutes on ice. The samples were centrifuged at a centrifugation rate of 12,000 g for thirty minutes to sediment the debris. A BCA Assay Kit (Solarbio Life Science, Beijing, China) was used in order to assess the protein content. Proteins (30*μ*g) were isolated using 10% SDS-PAGE and put into PVDF membranes. These plots were then subjected to incubation overnight at a temperature of 4°C with rabbit anti-*α*-SMA (1 : 500; Abcam), rabbit anti-S100A4 (1 : 1000; Abcam), rabbit anti-p-ERK1/2 (1 : 1000; Cell Signaling Technology), rabbit anti-fibronectin (1 : 1000; Abcam), rabbit anti-ERK1/2 (1 : 1000; Cell Signaling Technology), rabbit anti-FAK (1 : 1000; Cell Signaling Technology), rabbit anti-p-FAK (1 : 1000; Cell Signaling Technology), rabbit anti-p-GSK-3*β* (1 : 1000; Cell Signaling Technology), and rabbit anti-GSK-3*β* (1 : 1000; Cell Signaling Technology). As an internal control, GAPDH (1 : 10000; Abcam) was employed. After the membranes were washed, they were exposed to incubation for one hour at room temperature with HRP-conjugated secondary antibodies. The ImageJ program (version: 1.46) from the National Institutes of Health, Bethesda, MD, was utilized for the purpose of conducting the densitometric analysis.

### 2.10. Wound Healing Assay

Wound healing assays were carried out as previously described [32]. Briefly, 5 × 10^5^ L929 cells were cultured on a 90% confluence in a six-well plate, and a wound was inflicted by manual scraping with a 200 *μ*l pipette tip. The remnant cells were allowed to washing with PBS three times to remove the cellular debris. Then, the cells were treated with the M2-CM or recombinant S100A4 protein (0.5 *μ*g/ml) in a culture medium supplemented with 1% FBS. The control group was treated with RPMI-1640 containing 1% FBS. The lines were photographed and measured under a microscope at 0, 24, and 48 h postscratching.

### 2.11. Migration Assay

Transwell assays were performed for the purpose of assessing the migratory capacity of L929 cells. In summary, about 1 × 10^4^ L929 cells were suspended in 1% FBS media in the top chambers of a 24-well Transwell plate (pore diameter 8 *μ*m). Lower chambers were enriched with 10% FBS and (a) serum-free media, which was subjected to further incubation at 37°C in an incubator with 5% CO_2_ for 12 hours to maintain the same conditions as CM, (b) serum-free medium with S100A4 (0.5 *μ*g/ml), and (c) M2-CM. The Transwell plates were incubated for 12 hours, and cell migration was calculated according to the mean values of five random fields obtained from the lower surface of the filters.

### 2.12. Graphs and Statistics

The experimental data were analyzed and statistical graphics were created with the aid of Adobe Photoshop and GraphPad Prism 6. The findings are expressed in the form of the mean ± standard deviation. Prior to further investigation, the data were subjected to standardization. To examine substantial differences between the groups based on at least 3 independent experiments, the one-way analysis of variance (ANOVA) or Student's *t*-test was performed. The criterion of statistically significant difference was set as *P* < 0.05. All of the experiments were carried out at least three times.

## 3. Results

### 3.1. Primary Tumor-Induced Macrophage Recruitment and ECM Remodeling in the Premetastatic Lung

The commonest site of breast metastasis is the lung. To study the microenvironmental changes in the premetastatic lung, we built a lung metastasis model with 4T1 cells. 4T1 cells are a highly aggressive metastatic breast cancer cell line acquired from a spontaneously developing BALB/c mammary tumor [41]. It is a triple-negative breast cancer cell line that fails to express the human epidermal growth factor receptor 2 (HER2) gene, progesterone receptor (PR), and estrogen receptor (ER) [42]. The orthotopic implantation of 4T1 cells in the mammary fat pad, with the creation of primary tumors and subsequent metastatic expansion, resembles a variety of stages of malignant breast cancer, particularly spontaneous lung metastasis [43–45]. Days 0 to 14 were considered as the premetastatic phase in accordance with our earlier findings [37]. Breast cancer-bearing mice were used on the 14^th^ day following 4T1 cell inoculation in the following experiments, whereas the control group was injected with PBS rather than cancer cells.

**Figure 1:**
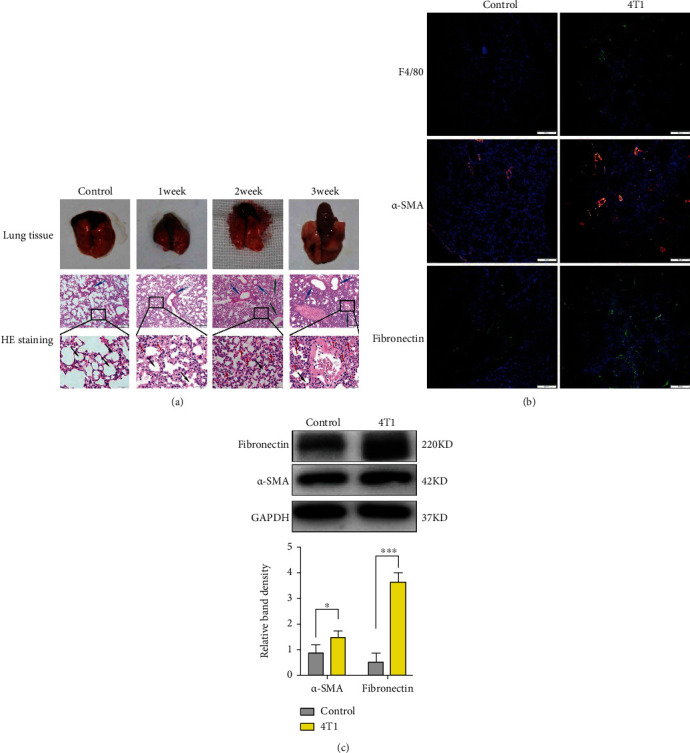
Primary tumor-induced macrophage recruitment and ECM remodeling in the premetastatic lung. (a) Morphology and H&E staining of breast cancer mouse lungs at 1 to 3 weeks (inflammatory cells: red arrows; blood vessel: blue arrows; bronchiole: green arrows; alveoli: black arrows). (b) Fluorescence microscopy showing the expression of F4/80, *α*-SMA, and fibronectin in control and breast cancer-bearing mouse lungs on the 14^th^ day. (c) Western blotting analysis showing that the levels of the ECM proteins *α*-SMA and fibronectin were markedly increased in tumor-bearing mouse lungs compared with control mouse lungs (^∗^*P* < 0.05, ^∗∗∗^*P* < 0.001).

In the premetastatic lungs, hematoxylin and eosin staining revealed accumulated inflammatory cells and an extensive stromal response (Figure 1(a) and Supplementary Figure 1). To further investigate the type of inflammatory cells accumulating at the premetastatic site, we analyzed the lung tissues by immunofluorescence techniques and found that the number of F4/80^+^ macrophages was increased in premetastatic lungs from tumor-bearing mice than that in controls (Figure 1(b)). These findings illustrated that the primary tumor could recruit macrophages to the premetastatic niche, which was in accordance with previous studies [7, 20, 46, 47]. In addition, the number of *α*-SMA^+^ myofibroblasts (key mediators of fibrosis), and the main ECM proteins, such as fibronectin, was also increased in premetastatic lungs from tumor-bearing mice (Figure 1(b)). Western blot analysis provided further evidence that the protein levels of *α*-SMA and fibronectin were markedly elevated in premetastatic lungs from tumor-bearing mice compared with control mice (Figure 1(c)).

### 3.2. Depletion of Macrophage Infiltration Inhibits Lung Fibroblast Activation and Metastatic Growth

**Figure 2:**
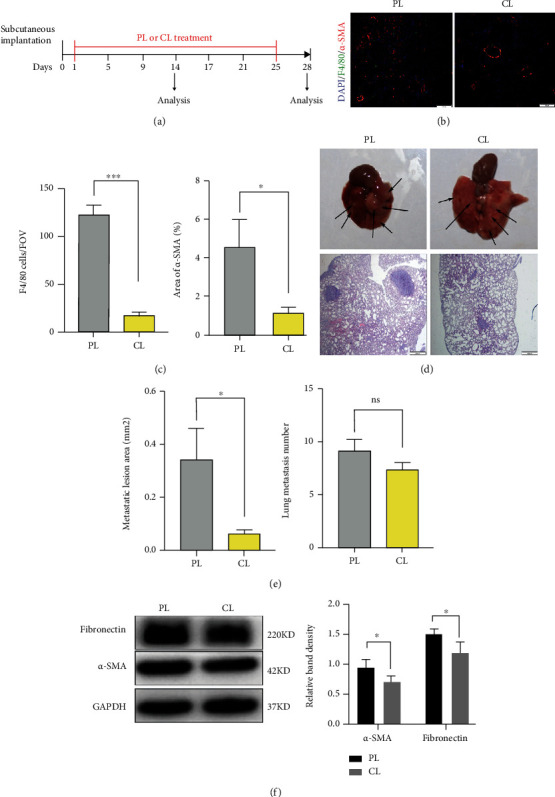
Depletion of macrophage infiltration inhibits lung fibroblast activation and metastatic growth. (a) Schematic illustration of the macrophage depletion experiment in vivo. (b) Representative immunofluorescence staining and quantification of F4/80^+^ macrophages and *α*-SMA^+^ myofibroblasts in premetastatic lungs from PL- or CL-treated mice. The nuclei are stained with DAPI. (c), Statistical analysis of the immunofluorescence staining for F4/80^+^ macrophages and *α*-SMA^+^ myofibroblasts (^∗^*P* < 0.05, ^∗∗∗^*P* < 0.001). (d) Metastases observed on the surface of tumor-bearing mouse lungs treated with PL or CL on the 28^th^ day. (e) Evaluation of metastatic frequency and lesions covered by metastatic cells in tumor-bearing lungs of mice treated with PL or CL (ns: not significant, ^∗^*P* < 0.05). (f) Protein levels of *α*-SMA and fibronectin in premetastatic lungs were measured by western blotting.

As previous studies reported, macrophages could promote metastasis not only by directly interacting with tumor cells but also by influencing other stromal cells [48–50]. We validated the infiltration of macrophages and *α*-SMA^+^ myofibroblasts in the premetastatic niche in the above results. For the purpose of investigating whether recruited macrophages are involved in lung fibroblast activation and tissue remodeling, we selectively depleted macrophages in vivo with clodronate liposomes (CL), a myeloid-specific ablating liposome that has previously been shown to promote the apoptosis of macrophage (Figure 2(a)) [38, 51]. As expected, in response to CL treatment, the number of F4/80^+^ cells was significantly reduced. Interestingly, we discovered that the number of *α*-SMA^+^ myofibroblasts in the premetastatic lungs of CL-treated mice was also decreased (Figures 2(b) and 2(c)). In addition, the expression of fibronectin and *α*-SMA proteins, which are the major markers underlying tissue remodeling, was also decreased in clodronate-treated mice (Figure 2(f)). Although the number of lung metastases was only modestly decreased in CL-treated mice, the size of the metastases was greatly reduced in response to macrophage depletion (Figures 2(d) and 2(e)). All of the above results suggested that macrophages are required for the activation of fibroblasts and metastatic growth.

### 3.3. M2 Macrophages Trigger Myofibroblast Activation through S100A4 Secretion

**Figure 3:**
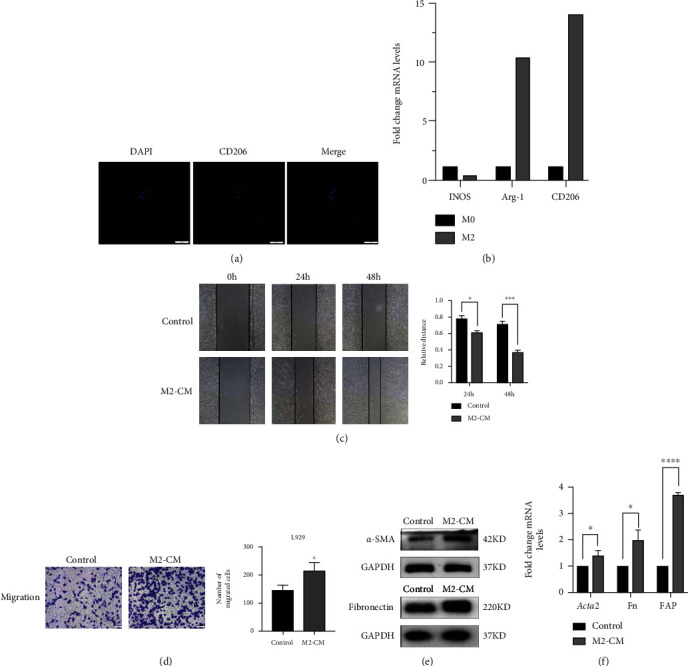
M2 macrophages induced migration and activation in L929 cells. (a) Immunofluorescence of polarization M2 macrophages. RAW 264.7 cells were polarized into M2 macrophages and labeled with anti-CD206. (b) M2 macrophage gene expression levels were quantified using qPCR. (c) Quantification of L929 fibroblast migration in the presence of control media or M2-CM by a wound-healing assay. (d) The migration ability of the control and M2-CM-treated groups was evaluated by Transwell assays. (e) L929 cells were treated with M2-CM for 48 h, and the protein levels of *α*-SMA and fibronectin were measured via western blotting. (f) Quantification of *α*-SMA (Acta2), FN, and FAP mRNA levels in fibroblasts stimulated with M2-CM, as determined by qPCR (^∗^*P* < 0.05, ^∗∗∗∗^*P* < 0.0001).

It has been reported that M2 macrophages have a close relationship with fibroblasts in the wound healing process. It can produce large amounts of cytokines, which promote myofibroblast formation from fibroblasts and subsequent production of collagen as well as excessive ECM deposition, leading to fibrosis [52, 53]. To investigate the role of macrophages in fibrotic pathology, we first stimulated macrophage polarization into M2 phenotypes using the IL-4 and IL-13 (Figures 3(a) and 3(b)). CM was harvested from M2 macrophages. Then, a scratch test and a Transwell assay were performed to investigate the effect of M2-CM on cell mobility. Compared with control cells, the L929 cells treated with M2-CM showed an increased migration ability (Figures 3(c) and 3(d)). In addition, incubation with M2-CM also promoted the activation of L929 cells, as confirmed by the expression of *α*-SMA, FN, and FAP (Figures 3(e) and 3(f)).

**Figure 4:**
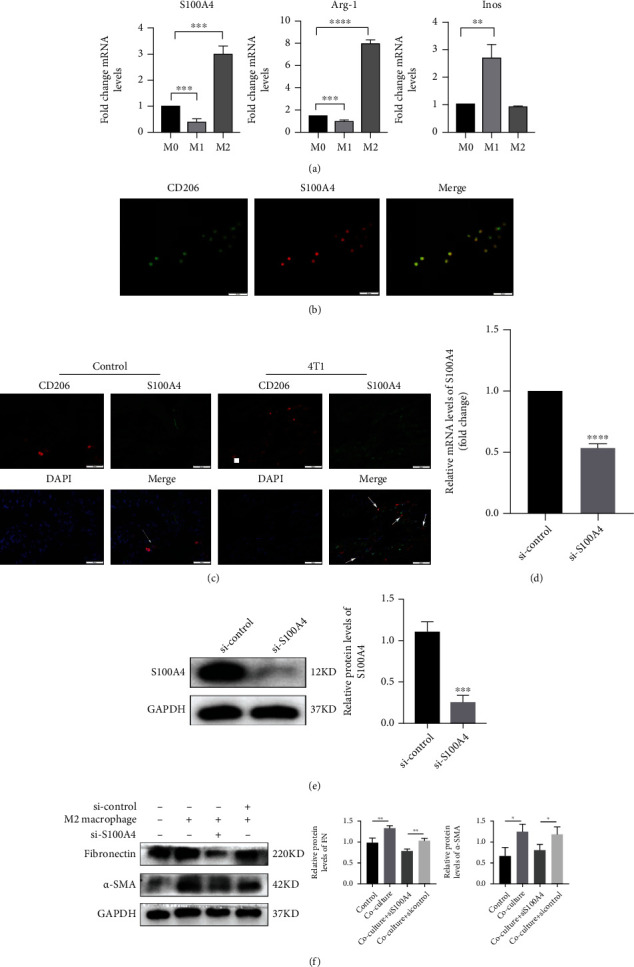
S100A4 is secreted by M2 macrophages and is correlated with fibroblast activation. (a) Expression gene profile of S100A4 in M0, M1, and M2 macrophages (^∗∗^*P* < 0.01, ^∗∗∗^*P* < 0.001, ^∗∗∗∗^*P* < 0.0001). (b) Double immunofluorescence showing that S100A4 colocalizes with CD206 in M2 macrophages. Yellow indicates colocalization of the two proteins. (c) Immunofluorescence costaining for S100A4 and the macrophage marker was performed on premetastatic lung sections of control and tumor-bearing mice. Arrows indicate the costaining. (d, e) The silencing efficiency of si-S100A4 assessed by qRT-PCR and western blot analysis. (f) M2 macrophages were transfected with control or S100A4 siRNA and then cocultured with L929 cells in a Transwell system. The expression of *α*-SMA and fibronectin in L929 cells was measured by western blotting.

Subsequently, we screened for potential factors that may be secreted from M2 macrophages and play a role in myofibroblast differentiation. We identified S100A4, a calcium-binding protein that is reported to play a crucial role in idiopathic pulmonary fibrosis by stimulating fibroblast migration and activation [32]. First, we observed that the expression profile of S100A4 matched the expression pattern of Arg1 in M2-polarized macrophages when compared with M0 and M1 macrophages (Figure 4(a)). S100A4 and CD206 colocalized in M2 macrophages, according to the results from double immunofluorescence staining (Figure 4(b)). We found an elevation of S100A4 protein in premetastatic lungs, which was in line with the in vitro data, and at least some of the S100A4 expression was localized to M2 macrophages (Figure 4(c)). M2 macrophages were subjected to transfection with S100A4 siRNA (control) and then coculturing with L929 cells in a Transwell system for forty-eight hours for the purpose of further studying the effect of S100A4 generated by M2 macrophages on the activation of normal lung fibroblast. The expression levels of S100A4 in the coculture system were determined by performing western blot analysis and qRT-PCR. The protein and mRNA levels of S100A4 were lower in the si-S100A4 group than those in the control group (Figures 4(d) and 4(e)). Compared with the coculture and si-Control-treated cells, we found that knockdown of S100A4 alleviated the effects of M2 macrophages on myofibroblast activation (Figure 4(f)). These findings suggest that S100A4 is one of the soluble factors secreted by M2-polarized macrophages, which can promote fibroblast activation.

### 3.4. S100A4 Promotes Lung Fibroblast Activation and Migration In Vitro

**Figure 5:**
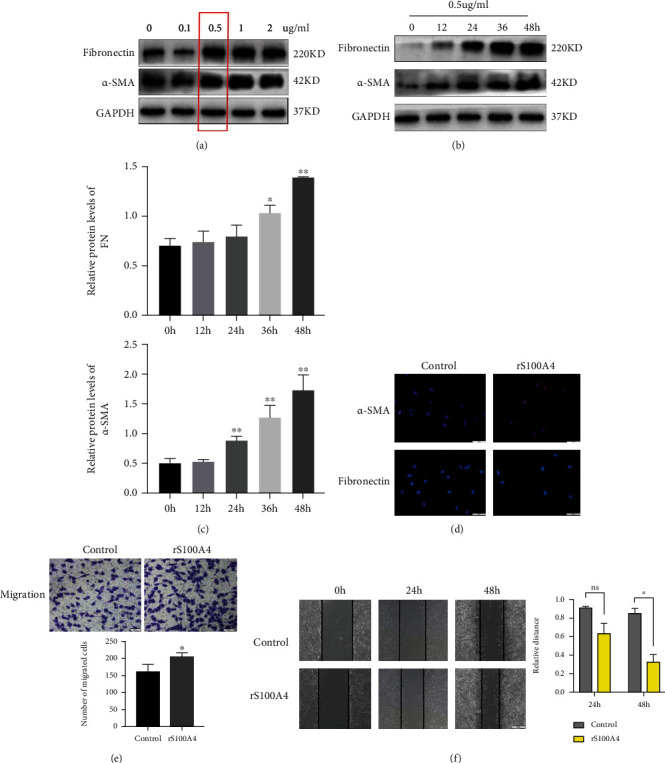
Extracellular S100A4 promotes lung fibroblast activation and migration in vitro. (a) L929 cells were treated with rS100A4 at different concentrations, and the protein levels of *α*-SMA and fibronectin were assessed via western blotting. (b) Cells were treated with rS100A4 (0.5 *μ*g/ml) for various periods of time, and the protein levels of *α*-SMA and fibronectin were measured via western blotting. (c) Densitometric and statistical analysis of western blots of the *α*-SMA and fibronectin content in fibroblasts after treatment with rS100A4 in western blot results (^∗^*P* < 0.05, ^∗∗^*P* < 0.01). (d),Fibroblasts treated with or without rS100A4 were stained for *α*-SMA (red) and fibronectin (green) by immunofluorescence; nuclei (blue) were stained with DAPI. (e) The migration ability of the control and rS100A4-treated groups was evaluated by Transwell assays (^∗^*P* < 0.05). (f) Direct migration of fibroblasts in the presence of 0.5 *μ*g/ml rS100A4 was analyzed by a wound-healing assay. Migration distances were measured by ImageJ (^∗^*P* < 0.05).

As we showed that F4/80^+^ macrophages in lung tissue express high levels of S100A4, we further investigated the influence of exogenous S100A4 on the activation of lung fibroblasts. We added the S100A4 cytokine to fibroblasts in a dose-dependent manner for twenty-four hours. The expression of fibronectin and *α*-SMA proteins increased significantly at 0.5 *μ*g/ml rS100A4, and this concentration was utilized in the following in vitro experiments (Figure 5(a)). Then, we evaluated the levels of *α*-SMA and fibronectin in fibroblasts after rS100A4 treatment in a time-dependent manner. As shown in Figures 5(b) and 5(c), the protein levels of *α*-SMA and fibronectin protein were elevated with time. Immunostaining provided further evidence that recombinant S100A4 could promote the activation of myofibroblasts characterized by high *α*-SMA and fibronectin (Figure 5(d)). Exogenous S100A4 has the capacity of directly stimulating lung fibroblasts, according to the findings of this experiment. Fibroblasts can migrate to the injured area and secrete a large amount of ECM to participate in tissue remodeling. After treatment with recombinant S100A4, the pulmonary fibroblasts showed an enhanced migration ability in the wound healing assay when compared with control cells (Figures 5(e) and 5(f)).

### 3.5. The ERK Signaling Pathway Is Involved in the S100A4-Induced Activation of Lung Fibroblasts

**Figure 6:**
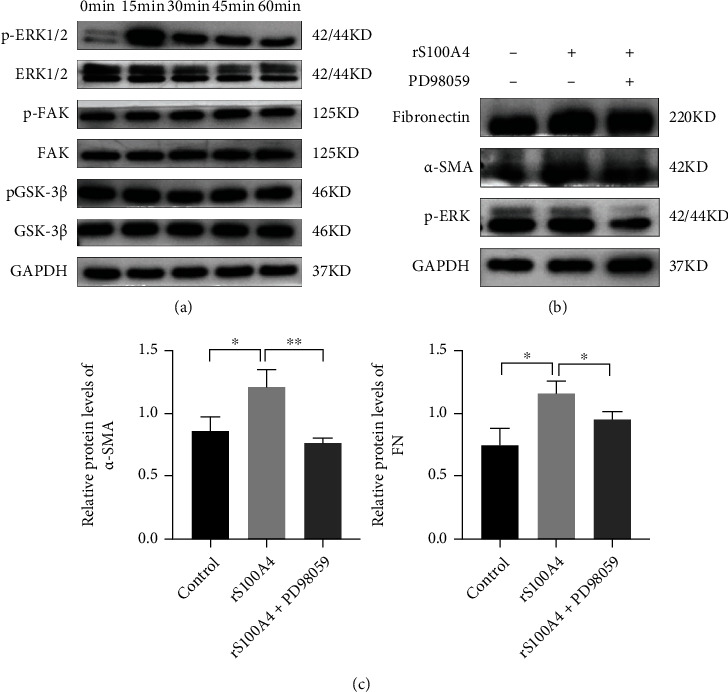
S100A4 promoted the activation of fibroblasts through ERK signaling. (a) L929 cells were treated with 0.5 *μ*g/ml rS100A4 for up to 1 h. Activation of p-ERK1/2, p-FAK, and p-GSK-3*β* (Ser9) was detected by western blotting analysis. (b) L929 cells were pretreated with PD98059 (20 *μ*mol/l) for 2 h, followed by 24 h incubation with or without rS100A4. Subsequently, the cells were collected, and the expression of *α*-SMA and fibronectin proteins was examined by western blotting analysis. (c) Densitometric and statistical analysis of the *α*-SMA and fibronectin content in fibroblasts in the western blotting results (^∗^*P* < 0.05, ^∗∗^*P* < 0.01).

To illustrate the molecular mechanisms of S100A4-induced activation of fibroblasts, we tested the common pathways that are involved in the incidence and progression of lung fibrosis. As shown in Figure 6(a), S100A4 rapidly induced Erk1/2 phosphorylation as early as 15 min. The FAK and WNT pathways were not activated in our studies with L929 cells. To further verify the function of ERK signaling in regulating S100A4-induced fibroblast activation, the ERK inhibitor PD98059 was added to the cell culture system before exposure to S100A4. According to the findings from the western blot analysis, the pharmacological inhibition of ERK blocked the S100A4-induced increase in *α*-SMA and fibronectin protein levels (Figures 6(b) and 6(c)). Collectively, these results demonstrated that the ERK signaling pathway is critical in mediating the S100A4-induced activation of fibroblasts and the expression of ECM proteins in L929 cells.

## 4. Discussion

In the present research, we gained a better comprehension of the role of macrophages in breast cancer metastasis with the aim of improving therapies for this deadly disease. In this regard, we discovered that macrophages aid breast cancer metastasis by secreting S100A4, which makes healthy lung fibroblasts become *α*-SMA^+^ myofibroblasts that release high levels of ECM proteins.

The remodeling of the ECM in the premetastatic niche plays a crucial role in metastasis development [11]. Previous research found that the remodeling of the ECM in the premetastatic niche facilitates the engraftment and early colonization of metastasizing cancer cells [12, 54]. Therefore, it is meaningful to identify the mechanism of ECM remodeling in the premetastatic niche. Many studies have reported an important role of primary tumor-derived exosomes and cytokines in modulating the premetastatic niche [9, 10, 20]. However, few studies have involved the role of normal stromal cells such as endothelial cells, inflammatory cells, and fibroblasts at the secondary metastatic site. In our study, we found substantial infiltration of monocyte-derived macrophages and *α*-SMA^+^ myofibroblasts in premetastatic lungs from tumor-bearing mice. Once macrophages are depleted of clodronate in vivo, the inhibition of macrophages is accompanied by the suppression of myofibroblast differentiation, which is the main cellular source of ECM proteins. Our results are in line with previous studies indicating that macrophages are involved in fibroblast activation, ECM remodeling, and lung metastasis [17, 18, 21]. However, the underlying cellular and molecular mechanisms are not well defined.

S100A4 was previously thought to be a protein expressed by fibroblasts. However, recent studies have shown that in idiopathic pulmonary fibrosis and liver fibrosis, its expression matches with the presence of macrophages [19, 31, 32]. Another study of pancreatic cancer liver metastasis also indicated that there were large amounts of secreted S100A4 proteins in macrophage culture supernatant in a secretome analysis [55]. Lung fibrosis can be induced by monocyte-derived macrophages [18, 56]. We hypothesized that the S100A4 derived from macrophages might also promote the development of ECM remodeling in the premetastatic niche. Our results are consistent with this hypothesis. In vivo study regarding immunohistochemistry staining of serial sections of premetastatic lungs showed colocalization of S100A4-positive cells and M2 macrophages. In addition, our in vitro experiments also showed the expression of S100A4 in macrophages. S100A4 exhibits both extracellular and intracellular properties. Within the cell, the presence of S100A4 plays an important role in numerous biological processes, such as apoptosis and cell differentiation [57, 58]. Here, we found that extracellular S100A4 could promote the activation of pulmonary fibroblasts and then promote the production of fibronectin in the lung. Our results are in line with prior studies showing that extracellular S100A4 promotes the lung fibroblast transition to myofibroblasts, which in turn synthesize ECM proteins with higher levels [19].

Many previous studies have confirmed that the ERK, WNT, and FAK signaling pathways play important roles in pulmonary fibrosis development [59–61]. To gain further comprehension into the molecular mechanisms pertaining to the S100A4-induced activation of fibroblasts and ECM remodeling, we examined the intracellular signaling pathways. Our results showed that S100A4 rapidly induced Erk1/2 phosphorylation, as early as within 15 min. However, the FAK and WNT signaling pathways were not activated. In addition, PD98059, as a pharmacological inhibitor of ERK, blocked the S100A4-induced increase in *α*-SMA and fibronectin levels in fibroblasts. Several S100A4-responsive cells have been shown to have the capacity of activating ERK1/2, including cardiac myocytes [35], pulmonary artery smooth muscle cells [36] rat hippocampus neurons [62], and human chondrocytes [63]. ERK1/2 is involved in their growth and survival responses and is thus a likely mediator of many of S100A4's effects. In addition, our studies indicated that the ERK signaling pathway also mediated the S100A4-induced activation of fibroblasts and the production of ECM proteins. However, there are still some shortcomings in our study. We hypothesized that both fibroblasts and macrophages can produce S100A4 and have a synergistic role. However, we did not test the basic expression of S100A4 in fibroblasts. The specific mechanism underlying this synergistic effect will be studied in our future research. In addition, mechanistic studies of S100A4 were conducted in cells but not in the premetastatic lungs. To validate these findings in vivo, more research in tumor-bearing animal models is required.

## 5. Conclusion

In summary, we identified that the macrophage-secreted S100A4 enhances the activation of fibroblasts via the ERK signaling pathway to facilitate ECM remodeling in the premetastatic niche. Intervention in the recruitment of macrophages or secretion of S100A4 may be promising for preventing the lung premetastatic niche formation.

## Data Availability

The data used to support the findings of this study are included within the article.

## Abbreviations

FBS: Fetal bovine serum
*α*-SMA: *α*-Smooth muscle actin
FAP: Fibroblast activation protein
TAM: Tumor-associated macrophage
LPS: Lipopolysaccharide
ATCC: American Type Culture Collection
ECM: Extracellular matrix
CL: Clodronate liposomes
PL: Pbs liposomes
CM: Conditioned media
PVDF: Polyvinylidene fluoride.

## Supplementary Materials

Supplementary Materials Supplementary Figure 1: histology score of pulmonary inflammation. (a) The score for perivasculitis. (b) The score for alveolitis.
